# Single-Nucleus Sequencing of an Entire Mammalian Heart: Cell Type Composition and Velocity

**DOI:** 10.3390/cells9020318

**Published:** 2020-01-28

**Authors:** Markus Wolfien, Anne-Marie Galow, Paula Müller, Madeleine Bartsch, Ronald M. Brunner, Tom Goldammer, Olaf Wolkenhauer, Andreas Hoeflich, Robert David

**Affiliations:** 1 Department of Systems Biology and Bioinformatics, University of Rostock, 18051 Rostock, Germany; 2 Institute of Genome Biology, Leibniz Institute for Farm Animal Biology (FBN), 18196 Dummerstorf, Germanybrunner@fbn-dummerstorf.de (R.M.B.);; 3 Reference and Translation Center for Cardiac Stem Cell therapy (RTC), Department of Cardiac Surgery, Rostock University Medical Center, 18057 Rostock, Germany; 4 Department of Life, Light, and Matter of the Interdisciplinary Faculty at Rostock University, 18059 Rostock, Germany; 5 Molecular Biology and Fish Genetics, Faculty of Agriculture and Environmental Sciences, University of Rostock, 18059 Rostock, Germany; 6 Stellenbosch Institute of Advanced Study, Wallenberg Research Centre, Stellenbosch University, 7602 Stellenbosch, South Africa

**Keywords:** snRNA-seq, RNA velocity, cluster analysis, cardiomyocytes, seurat

## Abstract

Analyses on the cellular level are indispensable to expand our understanding of complex tissues like the mammalian heart. Single-nucleus sequencing (snRNA-seq) allows for the exploration of cellular composition and cell features without major hurdles of single-cell sequencing. We used snRNA-seq to investigate for the first time an entire adult mammalian heart. Single-nucleus quantification and clustering led to an accurate representation of cell types, revealing 24 distinct clusters with endothelial cells (28.8%), fibroblasts (25.3%), and cardiomyocytes (22.8%) constituting the major cell populations. An additional RNA velocity analysis allowed us to study transcription kinetics and was utilized to visualize the transitions between mature and nascent cellular states of the cell types. We identified subgroups of cardiomyocytes with distinct marker profiles. For example, the expression of Hand2os1 distinguished immature cardiomyocytes from differentiated cardiomyocyte populations. Moreover, we found a cell population that comprises endothelial markers as well as markers clearly related to cardiomyocyte function. Our velocity data support the idea that this population is in a trans-differentiation process from an endothelial cell-like phenotype towards a cardiomyocyte-like phenotype. In summary, we present the first report of sequencing an entire adult mammalian heart, providing realistic cell-type distributions combined with RNA velocity kinetics hinting at interrelations.

## 1. Introduction

Single-cell sequencing allows for an in-depth characterization of complex tissues and their cell types [1]. However, there are two major issues when it comes to the cardiovascular system, namely, (i) the difficulty of dissociating the adult mammalian heart tissue without damaging constituent cells and (ii) technical limitations regarding cell capture techniques leading to an underrepresentation of individual cell types (i.e., cardiomyocytes) due to their large cell size and irregular shape [2]. Whereas research efforts aim to avoid these issues by relying on embryonic and neonatal murine hearts or focusing on non-myocyte populations in adult mouse hearts, we desisted from single-cell Ribonucleic acid sequencing (RNA-seq) and instead conducted single-nucleus RNA-seq (snRNA-seq), which has been shown to present similar transcriptomic results [3]. Currently, existing studies on adult mammalian hearts concentrate only on selected substructures such as the ventricle [4] or the conduction system [5]. To our knowledge, we present the first snRNA-seq analysis of an entire adult mammalian heart.

Recently, a method was established to predict even future states of individual cells using single-cell or single-nucleus data. The relative abundance of nascent (unspliced) and mature (spliced) mRNA in these datasets is exploited to predict the rates of gene splicing and degradation. The time derivative of the gene expression state is calculated on the basis of these gene splicing events and is referred to as RNA velocity [6]. The RNA velocity analysis of our snRNA-seq data allowed us to study transcription kinetics and revealed details about the dynamics and interconnectedness of our identified cell clusters.

## 2. Materials and Methods

### 2.1. Isolation of Nuclei

To avoid potential aberrations due to inbreeding, we relied on an outbred mice strain (Fzt:DU) [7]. Mice were handled in accordance with Directive 2010/63/EU on the protection of animals and with the Scientific Committee supervising animal experiments in the Leibniz-Institute for Farm Animal Biology (FBN), Dummerstorf, Germany. Whole hearts were harvested from 4 male mice (12 weeks) after cervical dislocation. The hearts were pooled and nuclei isolated using the Nuclei PURE Prep isolation kit (Sigma-Aldrich, Darmstadt, Germany) according to the manufacturer’s protocol. All work was carried out on ice. In brief, hearts were rinsed with ice cold PBS, minced thoroughly, and preincubated in 10 mL freshly prepared lysis buffer for 10–15 min before the tissue was further homogenized using a gentleMACS dissociator (Miltenyi Biotec, Bergisch Gladbach, Germany). Cell debris and clumps were removed by using 40 μm strainers. To purify the nuclei, lysate samples were mixed with 18 mL chilled sucrose cushion solution, layered on 10 mL pure 1.8 M sucrose cushion solution in a 50 mL Beckman ultracentrifuge tube, and centrifuged for 45 min at 30,000× *g* and 4 °C. Nuclei pellets were resuspended in 5 mL chilled PBS containing 1% BSA and 0.2 U/μL RNase inhibitor and cell debris was removed by a final filtration step. After centrifugation for 8 min at 600× *g* and 4 °C, the supernatant was carefully removed and nuclei were resuspended in 3 mL Nuclei PURE storage buffer. The samples were transferred to cryotubes, snap-frozen in liquid nitrogen, and stored at −80 °C until processing.

Sequencing was conducted by Genewiz (Leipzig, Germany) on the 10xGenomics system (Carlsbad, CA, USA). Single nuclei were captured in droplet emulsions and snRNA-seq libraries were constructed as per the 10x Genomics protocol using GemCode Single-Cell 3′ Gel Bead and Library V3 Kit (Carlsbad, CA, USA). RNA was controlled for sufficient quality on an Agilent 2100 Bioanalyzer system (Santa Clara, CA, USA) and quantified using a Qubit Fluorometer (Waltham, MA, USA). Libraries were subsequently sequenced on the NovaSeq 6000 Sequencing System (Illumina, San Diego, CA. USA).

### 2.2. Computational Data Analysis

The snRNA-seq fastq data files were aligned with kallisto (v.0.46) to the generated mm10 genome (Ensembl release 98) index. The UNIX source code containing the detailed steps of the generation is provided at our FairdomHub/iRhythmics instance (https://doi.org/10.15490/fairdomhub.1.study.713.1). Additionally, the latest version of the complete index build was shared at Zenodo for further reuse (https://doi.org/10.5281/zenodo.3623148). This index contains the spliced and unspliced transcript annotations of the mm10 murine needed for RNA velocity analysis. The kallisto alignment files were subsequently quantified with bustools (v.0.39.3) as previously described [8]. Subsequently, transcripts were integrated into R by using the BUSpaRse R-package (v.0.99.25) to be able to use the downstream processing tool Seurat (v.3.1.1). For clustering, dimensionality was initially reduced by principal component analysis and numbers of the most variable principal components were selected using heuristic methods implemented in Seurat. For an improved UMAP clustering and identification of small subgroups, we included the upstream processing algorithm harmony (v.1.0) [9]. The RNA velocity was conducted with the velocyto R-package (v.0.6) [6]. Sets of well-known marker genes were used to assign the underlying cell types of the generated clusters, as summarized in our computational R script. In addition, novel cell cluster markers recently identified by other groups working with single-nucleus data^4^ were applied and found to be transferable to our dataset.

The detailed experimental protocol, computational scripts, top 100 transcripts per cluster as well as the expression of the top markers for our identified clusters can be accessed from FairdomHub/iRhythmics. Raw data is provided in the Single Cell Expression Atlas via Arrayexpress (Accession ID: E-MTAB-8751).

## 3. Results and Discussion

**Figure 1:**
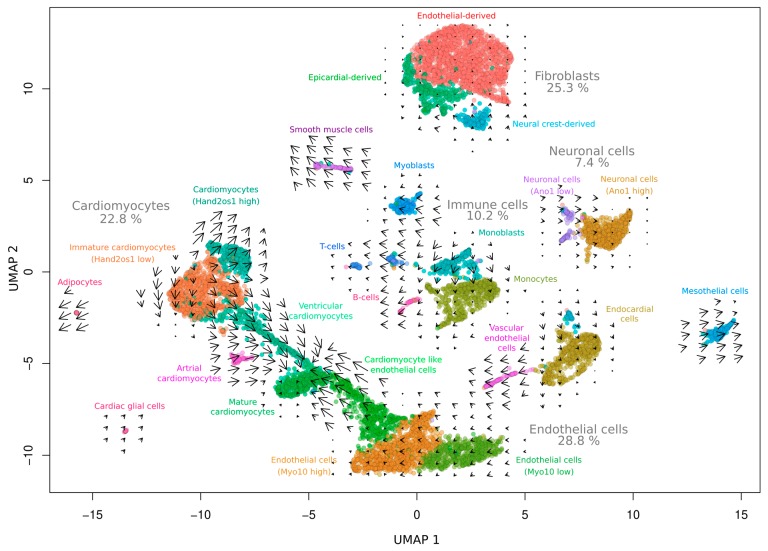
Single-nucleus transcriptome characteristics of pooled whole Fzt:DU mice hearts (*n* = 4). UMAP clustering of snRNA-seq data (8635 nuclei) reveals 24 distinct clusters for the indicated cell types. The arrows represent RNA velocity kinetics visualizing the direction and acceleration between mature and nascent mRNA. The percentages represent the nuclei ratio.

Single-nucleus analysis included a total of 8635 nuclei and 22,568 genes in which each cell exhibits an average total expression of 2662.6 reads. The analysis revealed 24 distinct clusters as a UMAP representation showing a global connectivity among the groups (Figure 1). The largest clusters can be attributed to populations of endothelial cells (28.8%), fibroblasts (25.3%), and cardiomyocytes (22.8%) containing ~2500, ~2200, and ~2000 nuclei, respectively.

**Figure 2:**
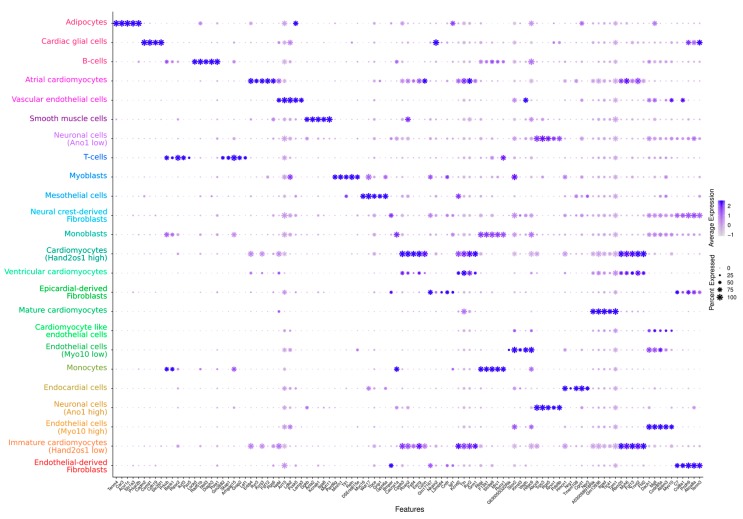
Dot-plot representation of the gene expression marker genes for the identified cell types. The size of dots represents the relative gene expression in percent for each cluster, e.g., a value of 100 means that each cell within this cell type expressed this gene. The color indicates the average expression level for the indicated gene per cell type. The color of the clusters is taken from Figure 1. A dot plot for the most significant gene per cluster as well as an extended visualization of the top 10 markers per cluster can be obtained at our FairdomHub/iRhythmics instance.

Interestingly, our data contradict earlier studies based on flow cytometry that suggest a much higher proportion of endothelial cells of up to 55% [10]. This disparity might be due to different isolation protocols, on the one hand, and the fact that we used whole hearts instead of isolated ventricles, on the other hand. However, more recent findings, also based on single-nucleus sequencing [5], are in accordance with our data, so that we further assume that this kind of holistic approach may yield more robust results than approaches relying on single-marker genes. Moreover, we not only observed various immune cells but also identified cells of neuronal origin (7.4%) and cardiac glial cells (0.2%) representing the innervated system of the heart and confirming the comprehensiveness of our data. The wealth of data enabled the identification of further cell-type markers that, in addition to the standard markers, facilitated the annotation of clusters, thereby providing novel reference points for us and the research community (Figure 2).

Our additional RNA velocity analysis of the snRNA-seq allowed us to study transcription kinetics (Figure 1). The indicated arrows show the direction and the velocity for future cell states. For example, immune cells undergo intense transformation processes upon maturation and activation and, therefore, show a high velocity (lengthy arrows) in our Fzt:DU mice. A quick turnover of RNA was also shown for smooth muscle cells, which have to adapt frequently to changing demands on the vascular pressure, confirming the physiological relevance of our data.

We furthermore identified subgroups of cardiomyocytes with distinct marker profiles and could visualize their developmental course by RNA velocity analysis (Figure 1). In particular, we found the expression of Hand2os1 [11,12,13]—a long non-coding RNA—that orchestrates heart development by dampening HAND2 expression, to distinguish immature cardiomyocytes from fully differentiated cardiomyocyte populations (Figure 2). Besides the mature, atrial, and ventricular cardiomyocytes, there is another Hand2os1 high cardiomyocyte population with a 1.5-fold expression enrichment apparently originating from the Hand2os1 low population. Based on very recent findings of de Soysa et al. [14], who identified *Hand2* as a specifier of outflow tract cells but not right ventricular cells during embryonal development, we assume that this population represents cells of the outflow tract.

Interestingly, mature cardiomyocytes appeared to originate not only from a single lineage but also from an additional endothelial direction (Figure 1). We found a cell population (cardiomyocyte-like endothelial cells) that comprises endothelial markers (e.g., Flt1, Dach1) as well as markers clearly related to cardiomyocyte function (e.g., Ryr2, Tpm1, Ttn, Gja1, and Myh6). The dual role of this population can also be recognized in the dot plot (Figure 2). Although the population lacked other typical cardiomyocyte markers (e.g., Tnnt2), together with the velocity data our results suggest a trans-differentiation process from an endothelial cell-like phenotype towards a cardiomyocyte-like phenotype, supporting previous findings [15].

As our data apparently include the findings of other studies, we are confident that our whole heart single-nucleus analysis of the outbred Fzt:DU mouse strain at present provides the most accurate representation of cell types in an adult mammalian heart and can be used as a reference for further comparative studies.
